# The Sendai river terraces monitored the co-seismic mega-thrusting

**DOI:** 10.1038/s41598-023-41031-6

**Published:** 2023-08-28

**Authors:** Soichi Osozawa, Hisatoshi Ito

**Affiliations:** 1 Institute of Geology and Paleontology, Faculty of Science, Tohoku University, Sendai, 980-8578 Japan; 2 Nuclear Risk Research Center, Central Research Institute of Electric Power Industry, Chiba, 270-1194 Japan; 3 Present Address: Kawaoso Molecular Bio-Geology Institute, Sendai, 982-0807 Japan

**Keywords:** Geodynamics, Geology, Geomorphology, Sedimentology, Seismology, Tectonics, Volcanology, Natural hazards

## Abstract

We conducted a detailed geological survey of the Sendai region, covering an area of 100 × 50 km. Our survey focused on accurately mapping river terraces, identifying the source volcanoes responsible for intercalated tephras, and locating the Nagamachi-Rifu fault and associated faults. The river terraces were observed and categorized based on their elevation relative to the present river channels. These terraces are predominantly found on the hanging wall of major reverse faults. Each terrace comprises fluvial gravels at the lower levels and eolian loam intercalated with local and regional tephras at higher levels, with the contact age corresponding to the time of emergence. To determine the ages of the terrace gravels, we employed a combination of zircon U–Pb dating, sedimentation rate calculations, and extrapolation techniques. This allowed us to establish the abandonment or emergent dates of the terraces. The formation of these terraces coincided with periods of fault activity, including hanging wall uplift, footwall subsidence, and fault vertical displacement, effectively monitoring the co-seismicity of the Nagamachi-Rifu fault. While we cannot predict the exact timing of future events, it is crucial to remain vigilant regarding the potential occurrence of a significant earthquake triggered by these fault activities.

## Introduction

The largest earthquakes in the world mostly take place along subduction zones, and these earthquakes have caused considerable damage and casualties throughout human history. In spite of the devastation wrought by great subduction zone earthquakes, more local seismic soures in the upper plate of subduction zones may pose greater hazard than more distant mega earthquakes to population and infrastructure located close to them. This study investigates stratigraphic–geomorphologic relationships of strath terraces in the Sendai area of northeast Japan to give insight into the Nagamachi-Rifu fault activity and its potential seismic hazard^1, 2^.

Both river (stream; fluvial) and marine (coastal) terraces are commonly believed to form through the intricate interplay of sedimentary, climatic, and tectonic processes. River terraces are formed by fluvial process, with the floodplain playing a significant role, and terrace risers forming parallel to the stream. In contrast, marine terraces result from wave activity, with the terrace scrape following the former strandline along the coast. Strath terraces, characterized by a bedrock surface higher than the modern stream channel bottom, are of particular interest, and some have proposed that they have form by the migration of knickpoints^3^. Knickpoints represent sections of a channel with relatively steep gradients situated between lower-gradient segments, and formed by variations in rock resistance, tectonic deformation, and base level changes. In alluvial streams, sediment transport rapidly smooths out irregularities in stream profiles through a combination of erosion in steep channel sections and downstream redeposition^4^. If strath terraces can be traced longitudinally, their formation may be attributed to the upstream migration of knickpoints and associated incision, unrelated to the phase of sea level fluctuation^5^.

Some recent studies on river terraces assign terrace ages directly to periods such as Marine Isotope Stage (MIS) 12a, which represents the date of a lowstand sea level during glacio-eustatic oscillations and base level falls, and similarly, MIS 5c is used to indicate a highstand sea level and base level rise^6^. For marine terraces, correlation with MIS 5e is commonly achieved by considering uniform rates of rock uplift, ranging from 0.9–1.2 mm/year^7^ to 1.2–1.6 mm/year^8^. Simplified conceptual or numerical models assume that terraces gradually and steadily rise above a base level, while bedrock rivers slowly incise into the landscape^9^.

Formation of strath terraces generally require rock uplift to raise strath elevations above the level of the present channel as the stream incises^10^. Flights of costal terraces are also generated as a result of rock uplift that raises a former coastal tread above present sea level^8, 11–13^. In some cases, such as the Himalaya^14^ and the Cascade Range^15^, the rock uplift rate may equal the incision rate associated with stream incision associated with strath terraces, whereas in other cases the incision rate may lag behind the rock uplift rate, such as in the Sierra Nevada of California^16^. Active tectonic uplift is a process also when driven by earthquakes, and the geomorphic consequences of such events are poorly understood and documented, except for the Mw = 7.6 Chi-Chi earthquake with vertical offsets of surface ruptures from ranged from 0.5 to 8 m^9^.

Strath terraces of the Sendai area were previously investigated by Nakagawa^17,18^ and Nakagawa et al.^19^. We sought to reexamine these terraces to provide new insight on regional seismic hazard. Through a comprehensive field survey, we found that the Sendai strath terraces are predominantly located on the hanging wall of the Nagamachi-Rifu reverse fault; the footwall is the Sendai coastal plain^1,2^. This observation suggests a transition from a fluvial to a terrestrial loam environment due to a sudden uplift of up to 100 m triggered by the Nagamachi-Rifu fault activity.

To determine the ages of the fluvial gravels, we employed a sedimentation rate of 0.01 mm/year for the overlying loam, considering the U–Pb dates of intersected tephras. Our findings suggest that the terrace gravel ages are not directly related to the marine isotope stages. Instead, the emergence of the terraces aligns with the periods of fault activity, thus serving as indicators of the cyclic yet intermittent mega-thrusting and simultaneous mega earthquakes. While we have gained a better understanding of past mega earthquakes, predicting the exact timing of future seismic events remains elusive, as the relationship between the age of terraces and the amount of uplift follows a regression line that lacks practical significance.

## Geological setting

The study area is located within the northeast Japan arc-trench system and provides insights into the geological history of the region. The older bedrock of the region records middle Cretaceous events, including the passage of a triple junction and the formation of the Futaba fault, originally considered a significant transform fault^20,21^. The older basement rocks in this area consist of middle Cretaceous granitic plutons, including adakite, which are partly found alongside pre-Silurian metamorphic rocks, particularly along the Futaba and related faults. Paleogene strata are scarce, and the Japan Sea's formation during the middle Miocene was coincident with the onset of terrestrial sedimentation and andesitic volcanism.

**Figure 1:**
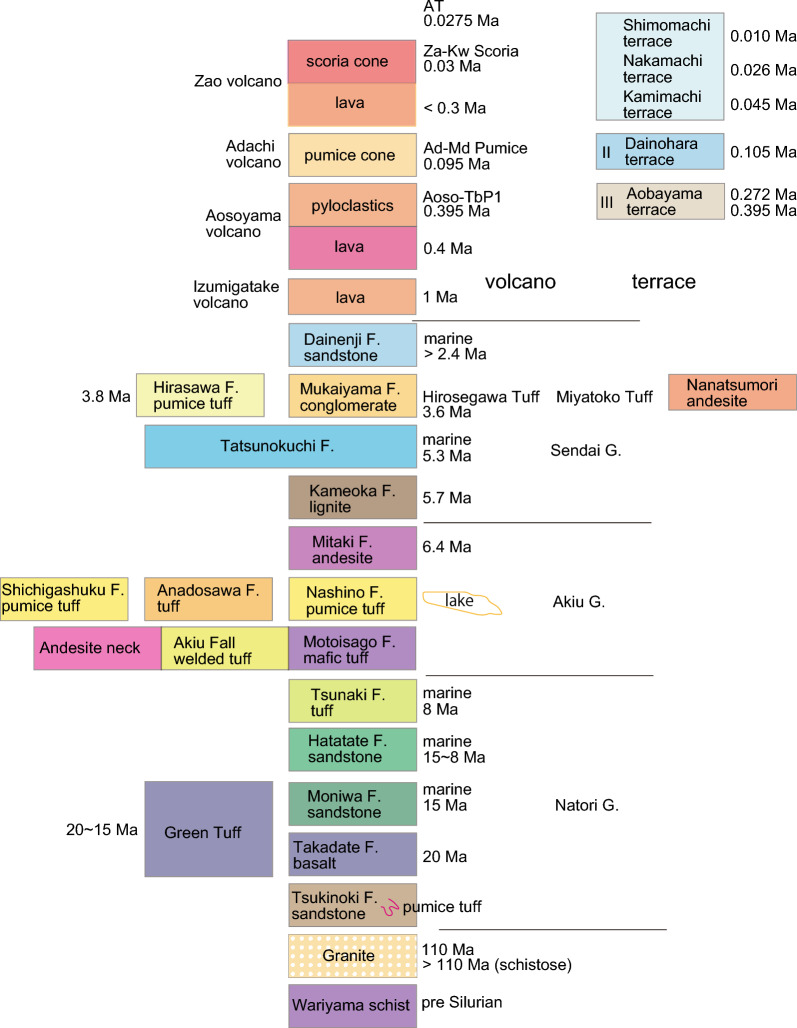
Summary of Sendai geology. Legend for regional geological map in Fig. 2.

**Figure 2:**
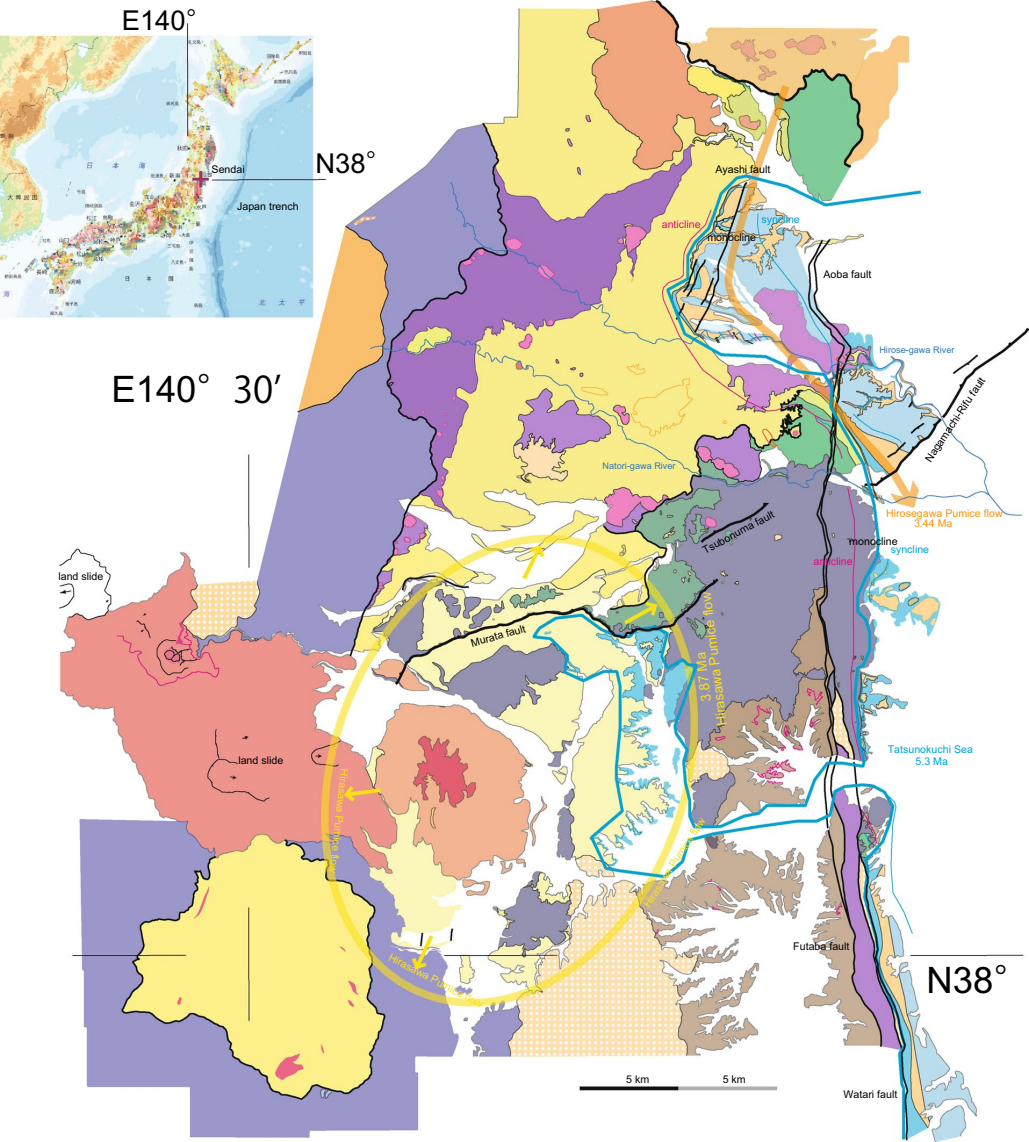
Pre Quaternary geological map of Sendai region. Sendai City area is located north of the Natori-gawa River. The geological map is original to the senior author, and was created using Adobe Illustrator CS3 (13.0.3). Inset: seamless geological map by geological Survey of Japan.

As the Japan Sea continued to open, marine sedimentation and basaltic volcanism occurred along the Japan Sea side and in areas between fragmented basement masses such as the Kitakami and Abukuma mountains. Over time, the marine environment gradually transformed into a shallow sea, eventually transitioning to terrestrial conditions characterized by caldera formation associated with development of andesitic necks, and caldera collapse by normal faulting in the late Miocene. During the Pliocene, terrestrial and shallow marine strata were deposited alternately over and adjacent to Miocene bedrock. The lower marine strata recorded the global marine transgression that followed the Messinian salinity crisis, leading to the formation of the Tatsunokuchi Sea^22^. The upper terrestrial strata encompass deposits associated with the Nanatsumori caldera, along with andesitic necks and the proto Aoso-yama volcano, which generated thick pyroclastic flows and ash falls. Detailed stratigraphy can be found in Fig. 1, the legend of Fig. 2, while the detailed geology is presented in our new geological map shown in Fig. 2. The Quaternary geology will be discussed in the following section.

## Quaternary volcanoes and related tephras (Fig. 3; Table 1)

**Figure 3:**
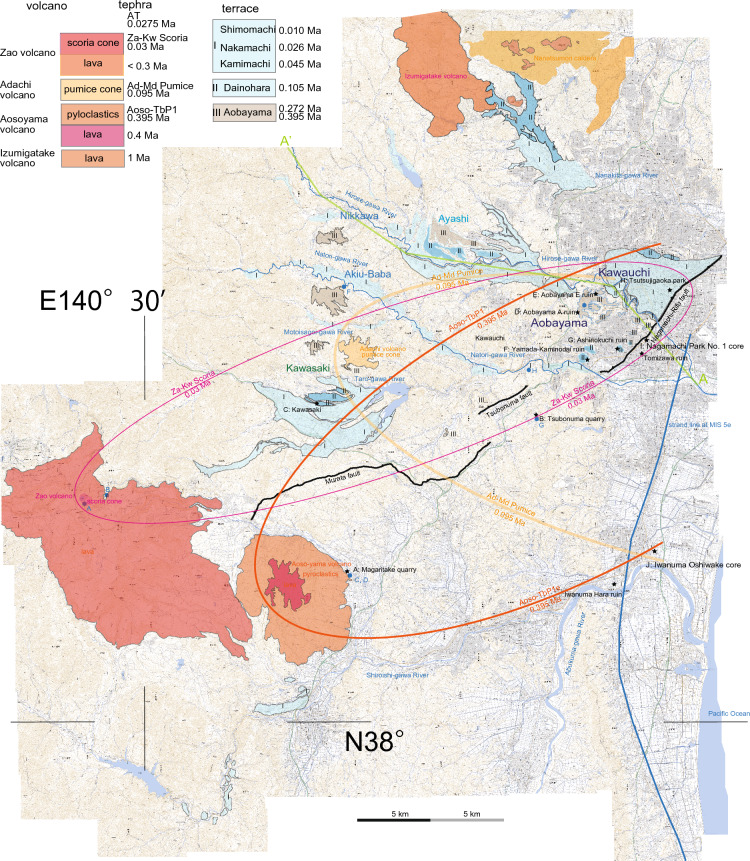
Terraces, local tepras, source volcanos, and reverse faults in the Sendai region. The geological map is original to the senior author, and was created using Adobe Illustrator CS3 (13.0.3). Ellipse shows distributional pattern of tephra and its source volcano. Background: digital topographic maps of 1: 25,000 scale, the Geospatial Information Authority of Japan.

**Table 1:** Local and regional tephras concerned to the present paper.

Tephra	Abbreviations	Ma	Reference for age estimate	Reference number
Aira-Tanzawa Ash	AT	0.0275	Machida and Arai (2003)	^33^
Zao-Kawasaki Scoria	Za-Kw	0.03	Itagaki et al. (1981)	^24^
Daisen-Kurayoshi Pumice	DKP	0.06	Machida and Arai (2003)	^33^
Aso-4 Ash	Aso-4	0.0875	Danhara et al. (2010)	^34^
Kikai-Tozurahara Ash	K-Tz	0.095	Machida and Arai (2003)	^33^
Ontake Pumice 1	On-Pm1	0.095	Aoki et al. (2008)	^35^
Adachi-Medeshima Pumice	Ac-Md	0.095	Present paper	
Toya Ash	Toya	0.106	Tomiya and Miyagi (2020); Ito (2014)	^36,37^
Adatara-Dake Ash	Ad-DK	0.12	Yamamoto (2012)	^38^
Hiuchigatake-Tagashira Ash	Hu-TG	0.129	Aoki et al. (2008)	^35^
Aoso-Tsubonuma Pumice 1	TbP1	0.395	Present paper	
Hirosegawa Tuff (Nanatsumori caldera)		3.6	Unpublished U–Pb age	
Hirasawa F. (proto Aoso-yama)		3.8	Unpublished U–Pb age	

See references in main text.

**Figure 4:**
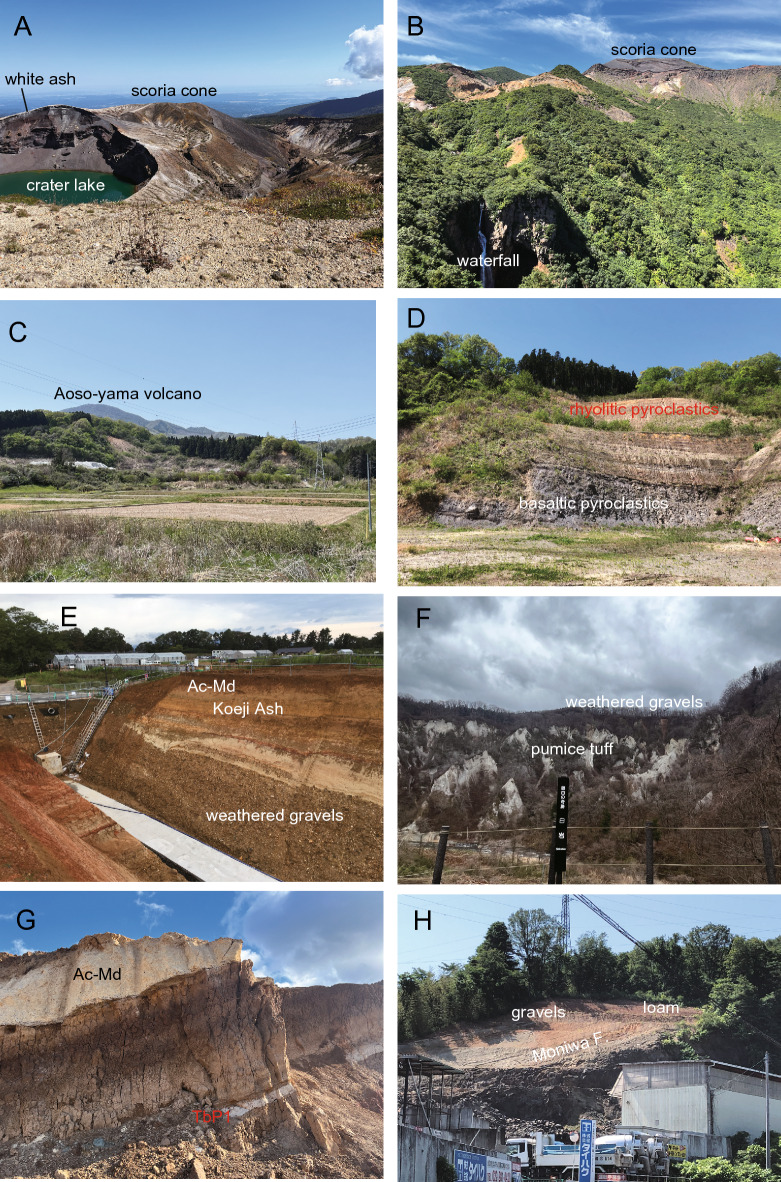
(**A**) The Zao volcano's crater lake is surrounded by a 3-m layer of white ash, which also covers the old crater. The blackish material present is scoria derived from the scoria cone. (**B**) The upper and lower lava flows of 0.3 Ma are accompanied by the 100-m Kaerazuno-taki waterfall. The sequence includes white altered lavas and pyroclastics, followed by block-colored, unaltered scoria cone at the summit. The Zao-Kawasaki Scoria (Za-Kw), dating back to 0.03 Ma, originated from this scoria cone. (**C**) Aoso-yama summits are primarily composed of lava, while the Magaritake quarry outcrop displays its lateral facies. (**D**) The quarry consists of 15-m basaltic pyroclastics, followed by 20-m dacitic pyroclastics, and finally 30-m rhyolitic pyroclastics which have weathered to a reddish color. The reddish layer at the top serves as the source of the Aoso-Tsubonuma Pumice 1 (TbP1). (**E**) The Aobayama II higher terrace, located near the "Nanno Terrace" in Aobayama, is observed at Tohoku University. The sequence from bottom to top includes weathered gravels, white fluvial silt, reddish "Koeji Ash", and the Adachi-Medeshima Pumice (Ac-Md). (**F**) The higher terrace can be found at the top of a 150-m outcrop and consists of 15 m of weathered gravels. The thick white pumice tuff belongs to the upper Miocene Nashino Formation. The observation was made from the lower terrace, with the present Natori-gawa River in the Akiu-Baba area. Sight seeing point named “white rock”. (**G**) Reddish loam is intercalated with a 50-cm layer of white Aoso-Tsubonuma Pumice 1 (TbP1), overlain by the 1.5-m of white Adachi-Medeshima Pumice (Ac-Md). In this photo, the contact with the basement rocks of the lower Miocene Takadate Formation, basaltic tuff breccia, can be seen. The outcrop is located at the top of the Tsubonuma quarry. (**H**) The Sendai Kamimachi terrace consists of a 1-m layer of lower gravels and a 1-m layer of upper loam. It unconformably overlays tilted black basaltic lava of the Takadate Formation and stratified shallow marine sandstone and conglomerate of the middle Miocene Moniwa Formation. This terrace is relatively downstream of the Natori-gawa River.

The currently active Zao volcano, features a crater lake (Fig. 4A). The volcano consists of two lava flows of ca. 0.3 Ma^23^, altered lavas and pyroclastics, and block colored, unaltered scoria cone, in ascending stratigraphic order (Fig. 4B). The crater lake formed at the summit of a scoria cone, that is covered by the youngest ash layer observed around the crater margin (Fig. 4A). The Zao-Kawasaki Scoria (Za-Kw) originated from this scoria cone and has been dated at 0.03 Ma^24^. The Zao volcano is characterized by andesitic and calc-alkaline compositions, and represents a volcanic arc front associated with ongoing subduction^25^.

**Figure 5:**
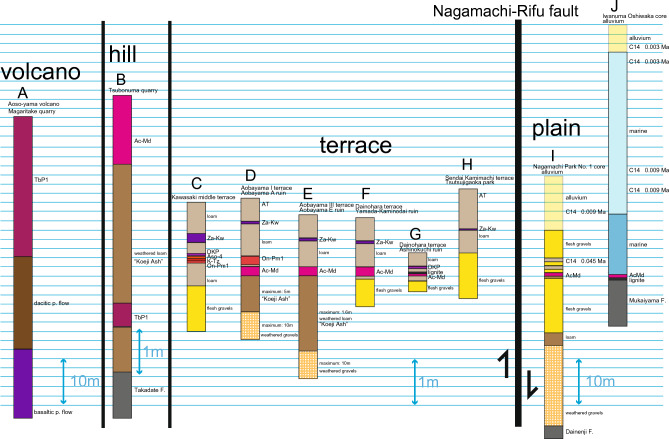
Illustrated columns showing distinct scales for the terrace (including the hill) and the plain (along with the volcano). The data presented in Table 3 primarily corresponds to the information gathered from these columns.

East of the Zao volcano, the Aoso-yama volcano, reactivated during the Pleistocene, exhibits tholeiitic volcanism, marking a trenchward shift of the arc volcanic front. This Pleistocene volcano (0.04 Ma; see below) consists of lava at the summit, and its lateral facies include basaltic pyroclastics, dacitic pyroclastics, and reddish-colored weathered rhyolitic pyroclastics, in ascending stratigraphic order (Figs. 4C,D, 5A). The uppermost reddish-colored pyroclastics constitute the Aoso-Tsubonuma Pumice 1 (TbP1^26^), known for its characteristic reddish color^27^.

The Adachi volcano is a pumice cone that erupted the Adachi-Medeshima Pumice (Ac-Md) eastward^28^. The Ac-Md pumice fall covers hills, terraces, and alluvial deposits (Fig. 5B–J). This pumice fall represents a single eruptive event dating at 0.095 Ma (see below). Unlike the Pliocene units found in the Aoso-yama volcano and the Nanatsumori caldera, the Adachi volcano lies oceanward of the present volcanic front^29,30^ and exhibits tholeiitic volcanism^31^.

Regional tephras, such as Toya Ash from Hokkaido, Ontake Pumice 1 from central Honshu (On-Pm1), Aso-4 Ash, Kikai-Tozurahara Ash (K-Tz) from Kyushu, and Daisen-Kurayoshi Pumice (DKP) from western Honshu, have been identified within loam overlying the Sendai area terraces (Fig. 5; Table 1; cf.^32^).

## Referenced dates of tephras (Table 1)

We determine the approximate age of the loam base or the terrace gravel top (= emergence time of terrace) by considering the sedimentation rate of the loam and referencing the known tephra ages. We had previously established the age of the Zao-Kawasaki Scoria (Za-Kw) as 0.03 Ma. Below we estimate the age of the Adachi-Medeshima Pumice (Ad-Md) and the Aoso-Tsubonuma Pumice 1 (TbP1).

Ad-Md Pumice: The age is well constrained based on dated regional tephras. At the Aobayama A ruin (Fig. 5D), the Ad-Md Pumice is directly overlain by the Ontake Pumice 1 (On-Pm1^28^). The age of the On-Pm1 tephra is estimated to be 0.0957 ± 0.0053 Ma^35^. The Kikai-Tozurahara Ash (K-Tz) also has an age of 0.095 Ma (cf.,^32^) and occurs above the On-P1 tephra^34^. Furthermore, the Ad-Md Pumice is overlain by the Aso-4 Ash (0.0875 million years ago^34^; approximately MIS 5b). However, the Toya Ash (0.106 million years ago^36^; Table 1; approximately MIS 5c) is absent. These dates indicate that the Ad-Md date is 0.095 Ma.

The Aoso-Tsubonuma Pumice 1 (TbP1) has not been dated, but we estimate its age based on its stratigraphic relationships to dated horizons as 0.395 Ma (Fig. 5B).

## U–Pb dating for TbP1 and Ad-Md tephras (Table 2)

**Table 2:** Zircon LA-ICP-MS U–Pb analytical results. Data in Italics (> 75% common Pb contamination) are excluded for further U–Pb analyses.

Sample name	Th (ppm)	U (ppm)	Th/U	*f*_Th/U_^a^	*f*_206_%^b^	Total^c^						Age [Ma]^d^		MSWD^e^	No	rho1	rho2
						^207^Pb/^206^Pb	2σ	^207^Pb/^235^U	2σ	^206^Pb/^238^U	2σ	^206^Pb/^238^U	2σ				
Medeshima tuff measured on 03/06/2023																	
MDS-7(2)	331	283	1.17	0.29	63.4	0.54461	0.13446	0.00553	0.00156	0.00007	0.00002	0.26	0.10		M07	0.75	0.56
MDS-13	33	57	0.58	0.14	65.0	0.55732	0.26869	0.01366	0.00766	0.00018	0.00008	0.51	0.47		M13	0.74	0.57
MDS-3	31	54	0.58	0.15	49.7	0.43681	0.21914	0.00787	0.00489	0.00013	0.00004	0.53	0.26		M03	0.89	0.59
MDS-19(2)	32	108	0.30	0.07	53.0	0.46275	0.65730	0.00483	0.00270	0.00008	0.00002	0.59	0.16		M19	0.74	− 4.53
MDS-5	11	26	0.45	0.11	64.9	0.55616	0.38130	0.02572	0.00976	0.00034	0.00016	2.26	1.06		M05	0.27	− 0.23
MDS-8	1123	961	1.17	0.29	6.3	0.09573	0.00891	0.01498	0.00114	0.00114	0.00006	6.95	0.36		M08	0.58	0.01
MDS-9	307	747	0.41	0.10	0.3	0.04860	0.00178	0.11272	0.00339	0.01683	0.00022	107.68	1.41		M09	0.80	− 0.33
MDS-12	405	721	0.56	0.14	0.3	0.04875	0.00143	0.11566	0.00388	0.01721	0.00032	110.11	2.01		M12	0.19	0.49
MDS-14	180	339	0.53	0.13	1.5	0.05768	0.01051	0.14833	0.02910	0.01866	0.00058	119.27	3.69		M14	− 0.20	0.51
*MDS-17*	*21*	*45*	*0.48*	*0.12*	*86.4*	*0.72524*	*0.33920*	*0.04314*	*0.01409*	*0.00043*	*0.00020*	*2.88 *	*1.28 *		*M17*	*0.79*	*0.34*
*MDS-18*	*48*	*81*	*0.60*	*0.15*	*88.4*	*0.74099*	*0.17783*	*0.02099*	*0.00700*	*0.00021*	*0.00005*	*1.42 *	*0.30 *		*M18*	*0.31*	*0.69*
*MDS-10(2)*	*42*	*67*	*0.63*	*0.16*	*92.5*	*0.77273*	*0.11504*	*0.06263*	*0.02028*	*0.00059*	*0.00016*	*3.88 *	*1.04 *		*M10*	*0.84*	*0.89*
*MDS-11*	*51*	*87*	*0.59*	*0.15*	*94.0*	*0.78445*	*0.68463*	*0.00642*	*0.00309*	*0.00006*	*0.00004*	*0.47 *	*0.25 *		*M11*	*0.40*	*− 0.20*
*MDS-1*	*40*	*74*	*0.54*	*0.13*	*96.8*	*0.80716*	*15.83737*	*0.01479*	*0.00597*	*0.00013*	*0.00004*	*0.95 *	*0.29 *		*M01*	*0.77*	*− 1415.17*
*MDS-2(2)*	*35*	*63*	*0.56*	*0.14*	*100.3*	*0.83461*	*2.30151*	*0.02066*	*0.00594*	*0.00018*	*0.00009*	*1.25 *	*0.58 *		*M02*	*0.97*	*− 25.23*
*MDS-4*	*47*	*77*	*0.61*	*0.15*	*105.7*	*0.87677*	*0.78614*	*0.00871*	*0.00286*	*0.00007*	*0.00004*	*0.56 *	*0.24 *		*M04*	*0.86*	*− 1.29*
*MDS-6(2)*	*19*	*37*	*0.52*	*0.13*	*110.2*	*0.91245*	*3.15123*	*0.04134*	*0.01157*	*0.00033*	*0.00012*	*2.21 *	*0.80 *		*M06*	*0.89*	*− 55.06*
*MDS-20*	*29*	*96*	*0.30*	*0.08*	*116.2*	*0.95927*	*0.88728*	*0.04660*	*0.01908*	*0.00035*	*0.00025*	*2.37 *	*1.60 *		*M20*	*0.89*	*− 0.33*
*MDS-15*	*13*	*28*	*0.45*	*0.11*	*133.5*	*1.09566*	*1.05862*	*0.07749*	*0.02917*	*0.00051*	*0.00025*	*3.40 *	*1.60 *		*M15*	*0.98*	*− 1.54*
*MDS-16*	*61*	*86*	*0.71*	*0.18*	*487.9*	*3.88052*	*3.10031*	*1.01851*	*0.91417*	*0.00190*	*0.00060*	*12.35 *	*3.88 *		*M16*	*0.59*	*0.47*
											Weighted mean (n = 4)	0.37	0.28	5.0			
Medeshima tuff measured on 03/23/2023																	
MDS-21(2)	138	293	0.47	0.12	0.9	0.05304	0.01221	0.00687	0.00136	0.00094	0.00006	6.11	0.35		No. 01	− 0.92	− 0.45
*MDS-22*	*19*	*41*	*0.45*	*0.11*	*104.7*	*0.86895*	*0.28251*	*0.10066*	*0.04326*	*0.00084*	*0.00029*	*5.51*	*1.86*		*No. 02*	*0.90*	*0.67*
*MDS-23*	*25*	*37*	*0.68*	*0.17*	*192.5*	*1.55892*	*2.21866*	*0.01204*	*0.00737*	*0.00006*	*0.00004*	*0.45*	*0.26*		*No. 03*	*0.75*	*− 1.45*
*MDS-24*	*13*	*23*	*0.55*	*0.14*	*92.2*	*0.77055*	*15.60260*	*0.04026*	*0.01144*	*0.00038*	*0.00017*	*2.54*	*1.09*		*No. 04*	*0.56*	*− 1620.68*
*MDS-25*	*24*	*39*	*0.60*	*0.15*	*98.9*	*0.82330*	*10.80740*	*0.00685*	*0.00509*	*0.00006*	*0.00004*	*0.48*	*0.26*		*No. 05*	*0.73*	*− 178.76*
*MDS-26(2)*	*20*	*36*	*0.55*	*0.14*	*108.9*	*0.90159*	*1.17802*	*0.03643*	*0.00989*	*0.00029*	*0.00012*	*1.98*	*0.75*		*No. 06*	*0.94*	*− 6.80*
*MDS-27(2)*	*20*	*37*	*0.53*	*0.13*	*99.0*	*0.82444*	*0.07579*	*0.55354*	*0.11523*	*0.00487*	*0.00103*	*31.42*	*6.61*		*No. 07*	*0.88*	*0.90*
*MDS-28(2)*	*54*	*79*	*0.68*	*0.17*	*94.6*	*0.78970*	*0.13282*	*0.08013*	*0.02567*	*0.00074*	*0.00020*	*4.83*	*1.26*		*No. 08*	*0.97*	*0.85*
*MDS-29*	*35*	*59*	*0.59*	*0.15*	*136.9*	*1.12198*	*0.37482*	*0.04603*	*0.02363*	*0.00030*	*0.00012*	*2.01*	*0.75*		*No. 09*	*0.99*	*0.76*
*MDS-30*	*19*	*28*	*0.70*	*0.17*	*105.1*	*0.87187*	*0.13734*	*0.39594*	*0.22647*	*0.00330*	*0.00172*	*21.30*	*11.05*		*No. 10*	*0.98*	*0.96*
*MDS-31*	*17*	*29*	*0.59*	*0.15*	*88.1*	*0.73852*	*0.28096*	*0.04478*	*0.01043*	*0.00044*	*0.00006*	*2.93*	*0.36*		*No. 11*	*0.20*	*− 1.28*
*MDS-32*	*18*	*34*	*0.52*	*0.13*	*80.3*	*0.67679*	*0.52599*	*0.02099*	*0.01107*	*0.00023*	*0.00007*	*1.55*	*0.43*		*No. 12*	*0.75*	*− 0.77*
*MDS-33*	*19*	*33*	*0.57*	*0.14*	*101.2*	*0.84119*	*0.06226*	*0.99323*	*0.46182*	*0.00857*	*0.00405*	*55.09*	*25.95*		*No. 13*	*0.98*	*0.99*
*MDS-34*	*25*	*45*	*0.55*	*0.14*	*78.8*	*0.66563*	*0.42226*	*0.02724*	*0.00787*	*0.00030*	*0.00012*	*2.01*	*0.77*		*No. 14*	*0.83*	*− 0.67*
*MDS-35*	*31*	*53*	*0.60*	*0.15*	*85.6*	*0.71894*	*0.22427*	*0.03133*	*0.00705*	*0.00032*	*0.00005*	*2.13*	*0.31*		*No. 15*	*0.73*	*− 0.35*
TbP1 tuff measured on 03/06/2023																	
TbP1-12	136	170	0.80	0.20	45.8	0.40606	0.12292	0.00589	0.00176	0.00011	0.00002	0.47	0.13		T12	0.97	0.33
TbP1-2	109	102	1.07	0.27	12.2	0.14172	0.04069	0.04894	0.01673	0.00251	0.00012	14.27	1.06		T02	0.97	1.11
TbP1-15	327	626	0.52	0.13	0.4	0.04929	0.00171	0.10651	0.00414	0.01568	0.00041	100.39	2.62		T15	0.79	0.48
TbP1-1	244	412	0.59	0.15	1.0	0.05381	0.00372	0.11935	0.00909	0.01609	0.00028	103.02	1.82		T01	− 0.55	0.49
TbP1-4(2)	529	570	0.93	0.23	1.4	0.05689	0.00249	0.13838	0.00480	0.01765	0.00052	112.87	3.29		T04	0.10	0.08
TbP1-14(2)	332	477	0.70	0.17	0.5	0.05029	0.00180	0.13161	0.00524	0.01899	0.00029	121.36	1.85		T14	0.92	0.44
*TbP1-10*	*83*	*91*	*0.91*	*0.23*	*87.4*	*0.73265*	*0.18113*	*0.08155*	*0.02962*	*0.00081*	*0.00023*	*5.29*	*1.51 *		*T10*	*0.95*	*0.74*
*TbP1-8*	*88*	*104*	*0.85*	*0.21*	*87.8*	*0.73604*	*0.11382*	*0.05991*	*0.01800*	*0.00059*	*0.00011*	*3.89*	*0.68 *		*T08*	*0.98*	*0.92*
*TbP1-5*	*371*	*274*	*1.35*	*0.34*	*91.8*	*0.76753*	*0.07269*	*0.07599*	*0.05115*	*0.00072*	*0.00042*	*4.70*	*2.70 *		*T05*	*0.99*	*1.00*
*TbP1-3*	*56*	*70*	*0.80*	*0.20*	*95.1*	*0.79341*	*0.14885*	*0.07975*	*0.01459*	*0.00073*	*0.00017*	*4.79*	*1.10 *		*T03*	*0.99*	*0.62*
*TbP1-9*	*67*	*78*	*0.86*	*0.21*	*97.6*	*0.81282*	*0.09293*	*0.12184*	*0.03368*	*0.00109*	*0.00027*	*7.09*	*1.76 *		*T09*	*0.97*	*0.91*
*TbP1-13*	*31*	*40*	*0.77*	*0.19*	*98.3*	*0.81897*	*0.13147*	*0.31251*	*0.07094*	*0.00277*	*0.00060*	*17.91*	*3.88 *		*T13*	*0.93*	*0.74*
*TbP1-11(2)*	*172*	*141*	*1.22*	*0.30*	*103.3*	*0.85791*	*0.17352*	*0.06864*	*0.01916*	*0.00058*	*0.00014*	*3.82*	*0.89 *		*T11*	*0.98*	*0.70*
*TbP1-7*	*155*	*134*	*1.15*	*0.29*	*104.8*	*0.86957*	*0.15606*	*0.07843*	*0.02268*	*0.00065*	*0.00015*	*4.29*	*0.96 *		*T07*	*0.98*	*0.78*
*TbP1-6(2)*	*54*	*68*	*0.80*	*0.20*	*111.5*	*0.92249*	*0.07379*	*0.21415*	*0.07168*	*0.00168*	*0.00053*	*10.94*	*3.39 *		*T06*	*0.97*	*0.97*
Bishop Tuff (reference age: 0.767 ± 0.001 Ma; Crowley et al., 2007) measured on 03/06/2023																	
BST-P31	1686	2773	0.61	0.20	16.3	0.17445	0.06350	0.00319	0.00161	0.00013	0.00002	0.81	0.11			0.89	1.07
BST-P32	912	1573	0.58	0.19	6.2	0.09487	0.01704	0.00160	0.00034	0.00012	0.00001	0.84	0.05			0.24	0.67
BST-P33	4103	4128	0.99	0.33	2.3	0.06408	0.01949	0.00100	0.00028	0.00011	0.00000	0.80	0.03			0.16	− 0.87
BST-P34	2097	3329	0.63	0.21	8.8	0.11547	0.04159	0.00188	0.00094	0.00012	0.00001	0.79	0.08			0.70	1.19
BST-P35(2)	2275	3344	0.68	0.23	10.8	0.13121	0.02412	0.00215	0.00032	0.00012	0.00001	0.78	0.04			0.28	− 0.65
Arithmetic mean	2215	3029	0.70	0.23							Weighted mean (n = 5)	0.80	0.02	1.0			
Fish Canyon Tuff (reference age: 28.402 ± 0.023 Ma; Schmitz and Bowring, 2001) measured on 03/06/2023																	
FCT20-90-2	263	394	0.67	0.30	2.1	0.06275	0.00467	0.04362	0.00363	0.00504	0.00019	31.85	1.19			0.87	0.45
FCT20-92-2	162	256	0.63	0.29	23.5	0.23046	0.04084	0.19851	0.04663	0.00625	0.00036	30.92	2.66			0.90	1.01
FCT20-93-2	555	595	0.93	0.42	0.2	0.04777	0.00283	0.03020	0.00151	0.00459	0.00007	29.53	0.48			0.01	− 0.47
FCT20-96-3	930	661	1.41	0.64	2.7	0.06699	0.00899	0.04203	0.00561	0.00455	0.00013	28.57	0.84			− 0.17	0.08
FCT20-97	454	672	0.67	0.31	0.2	0.04758	0.00285	0.03076	0.00208	0.00469	0.00014	30.22	0.90			0.13	0.47
Arithmetic mean	473	516	0.86	0.39							Weighted mean (n = 5)	29.70	1.20	5.7			
Plešovice (reference age: 337.13 ± 0.37 Ma; Sláma et al., 2008) measured on 03/06/2023																	
P3-1-1165-2	195	1556	0.13		0.8	0.05270	0.00047	0.39275	0.01303	0.05407	0.00158	339.58	9.69			0.82	0.97
P3-1-1166	120	967	0.12		0.8	0.05241	0.00110	0.38709	0.01080	0.05359	0.00110	336.64	6.76			0.14	0.66
P3-1-1167	120	1022	0.12		1.0	0.05373	0.00111	0.39532	0.01160	0.05339	0.00159	335.41	9.76			0.91	0.76
P3-1-1168	118	1006	0.12		0.8	0.05206	0.00082	0.39166	0.01398	0.05459	0.00133	342.76	8.17			0.75	0.93
P3-1-1169	111	948	0.12		0.9	0.05321	0.00171	0.40165	0.01804	0.05477	0.00132	343.84	8.11			0.87	0.72
P3-1-1170(2)	109	959	0.11		1.0	0.05358	0.00077	0.39455	0.00794	0.05344	0.00123	335.69	7.56			0.82	0.78
P3-1-1171	141	1196	0.12		1.5	0.05770	0.00187	0.42570	0.00929	0.05354	0.00122	336.30	7.53			0.00	− 0.05
P3-1-1172	131	1128	0.12		0.9	0.05304	0.00087	0.39440	0.00480	0.05395	0.00063	338.85	3.89			0.79	0.06
Arithmetic mean	131	1098	0.12								Weighted mean (n = 8)	338.6	2.3	0.6			

^a^*f*_Th/U_ = (Th/U)_zircon_/(Th/U)_magma_. (Th/U)_magma_ of 4.0 ± 2.0 for unknown, 3.0 ± 1.5 for Bishop Tuff, and 2.2 ± 1.1 for Fish Canyon Tuff were used.

^b^*f*_206_% denotes the percentage of ^206^Pb that is common Pb and is calculated as follows: *f*_206_% = 100 × (x − 0.0461)/(0.832–0.0461), where x is a measured ^207^Pb/^206^Pb ratio.

^c^Measured isotope ratio, that is before corrections of initial ^230^Th and ^231^Pa disequilibrium and common Pb.

^d^Individual U–Pb (^238^U–^206^Pb) ages were determined using the modified ^207^Pb method. Error of weighted mean is shown as 95% confidence level.

^e^MSWD: mean square weighted deviation.

^f^Statistical outlier identified by applying 2σ criterion in Isoplot.

The second author conducted U–Pb dating on two samples of the Aoso-Tsubonuma Pumice 1 (TbP1) and the Adachi-Medeshima Pumice (Ad-Md) collected by the senior author from the same outcrop at the Tsubonuma quarry (Figs. 4E, 5B). For detailed information on the U–Pb dating methodology, see Osozawa et al.^32^.

We observed contamination of common Pb (204Pb), likely due to intense weathering of the host eolian deposit (loam, reddish colored), in most of the Ad-Md samples. Consequently, data with contamination exceeding 75% were excluded from further analyses (Table 2; 11 out of a total of 20 samples had contamination > 75%). Based on the remaining four data points with younger ages for Ad-Md, we obtained a weighted mean age of 0.37 ± 0.28 Ma (95% confidence; n = 4; mean square weighted deviation = MSWD = 5.0).

As mentioned above, we estimated a much younger age of 0.095 Ma for the Ad-Md Pumice (Table 1), which we will employ for subsequent considerations. The discrepancy in ages may result one or combination of two causes. First, because of the high closure temperature of zircon (900 °C or more), zircon growth may significantly predate eruption (cf.,^32^); the pre-eruptive period of zircon growth becomes less significant for progressively older volcanic rocks. Second, it is possible that the Adachi volcano, which is the source of the Ad-Md Pumice, belongs to one of the most fore-arc side volcanoes characterized by island arc tholeiite, similar to the Aoso volcano^29–31^, indicating a genetic relationship between these volcanoes. The Aoso volcano has been dated at 0.4 Ma using the K–Ar method^39,40^. We have estimated the age of Aoso-TbP1 as 0.395 Ma.

## Stratigraphy of Late Quaternary terraces, hilly area, and fluvial plain (Fig. 3)

The Sendai terraces primarily occur along the Nanakita-gawa River, Hirose-gawa River, and Natori-gawa River, as well as their respective tributaries. However, the Abukuma-gawa River lacks significant terraces, except for smaller terraces along the upper stream of the branched Shiroishi River. The Sendai terraces can be defined as occupying different relative elevations above streams, including the higher and lower temperate Aobayama terrace (marked as III in Fig. 3), the middle and higher temperate Dainohara terrace (II), and the lower and lower temperate Kamimachi, higher temperate Nakamachi, and Shimomachi terraces (I), as described by Nakagawa et al.^19^.

The Aobayama terrace deposits are composed of fluvial gravels overlain by a layer of loam (Fig. 4E). The gravel layer represents floodplain deposits from ancient rivers such as the Hirose-gawa River, Natori-gawa River, and their tributaries. The loam consists of aeolian deposits intercalated with local tephras, including the Ac-Md and Za-Kw, as well as regional tephra such as On-Pm1 (Fig. 5D,E). The lower but main portion of the loam, situated below the Ac-Md layer, is characterized by a reddish weathering color and is referred to as "Koeji Ash" (191,960). The TbP1 pumice is reported to be absent in the "Koeji Ash" according to Otsuki^26^. Higher terraces can be observed upstream of the Hirose-gawa River, Natori-gawa River, and their tributaries (Fig. 4F).

The TbP1 pumice is present in hilly/upland areas, including valleys, where it is intercalated within reddish-colored weathered loam known as the "Koeji Ash" (Figs. 4G, 5B). The Ac-Md pumice is similarly found in upland areas, partially associated with the "Koeji Ash" (Figs. 4G, 5B), covering the basement relief beyond the Aobayama terrace (Fig. 4E). The late Pleistocene loam covered the upland area, almost conforming the current landscape.

The Dainohara terrace, classified as a middle terrace, consists of unweathered gravels overlain by loam associated with Ac-Md and Za-Kw deposits (Fig. 5F,G). The Sendai Kamimachi terrace, categorized as a lower terrace, is associated with the Za-Kw deposit but lacks the Ac-Md layer (Figs. 4H, 5H). The Sendai Nakamachi and Shimomachi lower terraces is loam poor and consists primarily of gravel.

The Nagamachi Park No. 1 core, located at an altitude of 20 m, lies on the footwall of the Nagamachi-Rifu fault at the western end of the Sendai Plain (Fig. 3). The occurrence of Ac-Md pumice is observed within thick Pleistocene fluvial gravels (Fig. 5I). The gravels below the Ac-Md layer exhibit contrasting weathering compared to those above it and are intercalated with loam that is comparable to the "Koeji Ash" (Fig. 5I). The top sequence, measuring 12 m in thickness, consists of alluvium, and the gravels and sands between the alluvium and the Ac-Md deposit are correlated with the gravels found in the lower terraces (Fig. 5I) and have a radiocarbon age of 0.045 Ma (Fig. 5I).

The Sendai Plain is predominantly composed of alluvium, characterized by basal gravels that have been deposited since the Younger Dryas period (0.0117 Ma; Holocene). In the middle horizon, there is a shallow marine lens, whereas the upper layers consist mainly of fluvial gravels, sands, and silts^41,42^. The thickness of the alluvium exceeds 50 m, and the paleo-channels of the Nanakita-gawa, Hirose-Natori gawa, and Abukuma-gawa rivers are buried to depths ranging from minus 60 to 80 m^41,42^. The basement underlying the Sendai Plain is predominantly composed of Pliocene strata, with the exception of specific areas such as the Nagamachi pool core where Pleistocene deposits are present above the Pliocene basement. Another exception is the Iwanuma Oshiwake core^43^ (Fig. 3), where fragmented Ac-Md pumice is found between the Pliocene basement and the Holocene shallow marine deposits (with the basal gravels of the Holocene being absent) (Fig. 5J). This occurrence of Ac-Md pumice represents its easternmost extent beneath the Sendai Plain, similar to the aforementioned Nagamachi pool core.

## Sedimentation rates and terrace chronology (Table 3)

**Table 3:** Estimation of sedimentation rates, tephra ages, and terrace gravel ages (= terrace emergent ages).

	Column	Lower tephra	Age Ma	Upper tephra	Age Ma	Thickness between tepras cm	Rate mm/year (***loam*** fluvial)	Loam thickness (maximum) cm	Terrace gravel age Ma	Terrace alluvium	References	Reference number
Aobayama A ruin	Figure 5D	On-Pm1	0.095	Za-Kw	0.03	70	***0.010***	300	**0.395**	III Aobayama I and II	Sendai City Board of Education (1990)	^44^
Aobayama E ruin	Figure 5E	Ac-Md	0.095	Za-Kw	0.03	60	***0.009***	160	**0.272**	III Aobayama III and IV	Archaeological Research Center on the Campus, Tohoku University (2001)	^45^
Yamada-Kaminodai ruin	Figure 5F	Ac-Md	0.095	Za-Kw	0.03	50	***0.008***	10	**0.108**	II Dainohara	Sendai City Board of Education (2003)	^46^
Ashinokuchi ruin	Figure 5G	Ac-Md	0.095	Soil	0	100	***0.011***	10	0.105	II Dainohara	Archaeological Research Center on the Campus, Tohoku University (2001)	^45^
Kawasaki	Figure 5C	Aso-4	0.0875	Za-Kw	0.03	60	***0.010***	50	0.133	II	Hataya (2005)	^47^
Adachi		Aso-4	0.0875	Za-Kw	0.03	80	***0.010***	50	0.133	Ac-Md	Yagi & Hayata (1989)	^48^
Sendai Kamimachi		Peat	0.031	Soil	0	180	***0.058***	150	0.056	I Sendai Kamimachi	Takeuchi (1986)	^49^
Sendai Kamimachi	Figure 5H	Za-Kw	0.03	AT	0.0275	30	***0.120***	60	0.350	I Sendai Kamimachi	Kosaka et al. (2014)	^50^
Nagamachi Park No. 1 core	Figure 5I	Peat	0.045		0		0.444		**0.045**	I Sendai Kamimachi	Miyagi Prefecture (1997)	^51^
Sendai Nakamachi		Peat	0.026		0	100	***0.038***	0	**0.026**	I Sendai Nakamachi	Itagaki et al. (1981)	^24^
Nagamachi Park No. 1 core	Figure 5I	Peat	0.009		0	800	0.888		**0.010**	I Sendai Shimomachi	Miyagi Prefecture (1997)	^51^
Nagamachi Park No. 1 core	Figure 5I	Ac-Md	0.095		0	2000	0.210			Alluvium	Miyagi Prefecture (1997)	^51^
Iwanuma Oshiwake core	Figure 5J	Ac-Md	0.095		0	3750	0.394			Alluvium	Kanisawa and Takeuchi (1997)	^43^
Iwanuma Hara ruin		Pottery	0.0014		0	100	0.740			Alluvium	Iwanuma City Board of Education (2020)	^52^
Tomizawa ruin		Peat	0.023		0	700	0.304			Alluvium	Sendai City Board of Education (1989)	^53^
Tomizawa ruin		Peat	0.024		0	400	0.166			Alluvium	Sendai City Board of Education (2004)	^54^
Iwakiri core		Peat	0.0067	Peat	0.0023	1000	2.270			Alluvium	Awata (2003)	^55^
Iwakiri core		Peat	0.008		0	1000	1.250			Alluvium	Awata (2004)	^56^

See references in main text.

Significance of bold-italic values: Rate mm/year for loam; significance of underlined values: Rate mm/ year for fluvial; significance of bold values: Adopted terrace gravel age Ma.

Depositional changes from fluvial to aeolian sedimentation provide valuable information about the emergence of previous floodplains. By fixing the ages of the Ac-Md and Za-Kw tephras, we can estimate the sedimentation rate of the loam layer located between these two volcanic deposits, taking into account its known thickness (Fig. 5; see^32^ for more details). The next step involves estimating the basal age of the loam layer, again considering its known thickness and applying the previously obtained sedimentation rate. These calculations, including the estimated sedimentation rates and the ages of terrace gravels (equivalent to the basal age of the loam layer and representing the emergence time of the terrace), are summarized in Table 3.

The sedimentation rate of terrace gravels exceeds 0.21 mm/year (with a maximum of 2.27), which is significantly higher compared to the sedimentation rate of loam, mostly around 0.01 mm/year (with an exceptional maximum of 0.12). This stark difference in sedimentation rates contributes to the relatively thin nature of the loam layer. In hilly areas covered by Pleistocene loam, the original morphology has been well-preserved. Conversely, due to the higher sedimentation rates, gravel layers are approximately ten times thicker in the Sendai coastal plain (as seen in cores I and J, which are scaled differently in Fig. 5). Additionally, the Aoso-yama column A in Fig. 5 exhibits a considerable thickness, reflecting the high sedimentation rate of the volcanic succession (cf.,^32^).

## Rapid emergence and intermittent uplift of terraces

The formation of each terrace did not occur gradually but rather intermittently at specific time intervals, as estimated above. The higher terraces emerged at approximately 0.395 and 0.272 Ma, the middle terraces at 0.108 Ma, and the lower terraces at approximately 0.045, 0.026, and 0.010 Ma (Table 3). It is important to note that the date of 0.108 Ma for the Dainohara middle terrace corresponds to Marine Isotope Stage (MIS) 5c as assigned by Toyoshima et al.^27^, rather than MIS 5e. The fact that the Aobayama higher terrace occupies consistently higher elevations than the Dainohara middle terrace, and the middle terrace consistently higher elevations than the lower terraces, provides evidence of intermittent rock uplift occurring at the aforementioned dates. We do not consider gradual uplift as the mechanism for the emergence of the Sendai terraces.

The Dainohara terrace, estimated to have emerged at 0.108 Ma (MIS 5c) as discussed above, was previously correlated with the Shimosueyoshi terrace in the Tokyo and Yokohama areas^17^. The Tokyo-Yokohama terrace is a marine terrace likely formed during MIS 5e, and the overlying Kanto loam contains the On-Pm1 tephra, similar to the Aobayama I terrace (Fig. 5D), along with other older tephras. The Shimosueyoshi terrace, located 5 km inland from the present Yokohama shoreline, experienced rapid emergence from west to east within a timeframe of 100–1000 years^57^, indicating punctuated uplift and emergence rather than gradual regression.

Along the Fukushima coast, approximately 50 km south of the mapped area, lies the Tsukabara Formation, a shallow marine terrace associated with the transgression during MIS 5e^58^. The strandline, located 1 km away from the present shore, is parallel to the current Fukushima coast^59^. The Tsukabara Formation consists of basal marine gravels-sands intercalated with the Hiuchigatake-Tagashira Ash (Hu-TG; 0.129 Ma) and top cross-stratified gravels-sands, which are also marine in origin and covered by terrestrial loam associated with the Adatara-Dake Ash (Ad-DK; 0.12 Ma) (see Table 1). The outcrop, observed in a 15 m high sea cliff, indicates that no significant uplift has occurred since MIS 5e there.

Additionally, Pleistocene shallow marine deposits related to the Ac-Md Pumice have been identified in the Iwanuma Oshiwake core (Figs. 3, 5J), with the MIS 5e (or MIS 5c) transgression recorded below the alluvium^43^. In this particular case, the strandline may have subsided and formed a buried terrace (Fig. 3).

Marine terraces are commonly observed on the Muroto Peninsula, Shikoku^8^. One of the higher terraces on Muroto Peninsula is correlated with MIS 5e, while one of the lower terraces is correlated with MIS 5c, based on the presence of K-Tz cover (cf., Table 1). The uplifting and northwestward tilting of the terraces can be attributed to the activity of the Muroto-Misaki Fault offshore, with additional minor faults intersecting the Muroto terraces.

## Quaternary monoclines, reactivated transcurrent fault, and reverse faults (Fig. 2)

Caldera-collapse normal faults, known as ring faults, formed during the Miocene period^60^. No other faults have been observed in Miocene and Pliocene strata, except for the Cretaceous Futaba transform fault and its associated strands (Fig. 2). The prevailing stress regime since the beginning of the Quaternary is characterized by east–west compression. Under this current stress regime, the Miocene caldera collapse normal faults have been reactivated as reverse faults, and some of these faults have generated damaging earthquakes^61,62^.

A distinct north–south trending monocline marks the boundary between Miocene and Pliocene strata, effectively delineating the paleo-shoreline of the Tatsunokuchi Sea (Fig. 2). In this figure, the monocline is defined by the axes of anticlines and synclines (cf.,^63^). All the terraces horizontally overlie Miocene-Pliocene strata, which are tilted within the monocline^64^. The monocline formed during the early Pleistocene, after the deposition of the Pliocene strata, and before the emergence of the Aobayama terrace (0.395 Ma).

The north–south striking transcurrent faults, collectively known as the Aoba fault as a northern extension of the Futaba fault, intersect the monocline as well as the tilted Miocene and Pliocene strata. These faults also cut Pleistocene horizontal terraces, including the lower terrace of Sendai Nakamachi^65^.

Three distinct reverse faults can be traced in an ENE–WSW trend, although they are not connected to each other (Fig. 3). The N–S trending Aoba transcurrent fault intersects both the Nagamachi-Rifu fault and the Tsubonuma fault (Fig. 2). While the crosscutting relationship is not observed, it is likely that these reverse faults were formed concurrently under a NW–SE compressional stress regime.

The terraces are on the hanging wall of the Nagamachi-Rifu fault, as indicated in columns D to H, whereas the footwall is the Sendai coastal plane, represented by the Nagamachi pool core column I in Fig. 5. Consequently, the fault delineates and crosscuts the southeastern terraces as well as the northwestern plain. Notably, all the Sendai terraces are consistently situated on the hanging wall of the Nagamachi-Rifu fault, in addition to the Tsubonuma and Murata faults (Fig. 3). No terrace gravels were observed along the northwestern margin of the Sendai plain, where the Ac-Md Pumice and underlying loam directly overlie Pliocene basement. Lower terraces are present along the upper reaches of the Shiroishi-gawa River, unrelated to the Nagamachi-Rifu fault.

The NNE–SSW-striking Ayashi fault crosscuts the higher and lower terraces. This fault is associated with back thrusts that also cut the terraces^66^.

The formation of the monocline, reverse faults, and transcurrent faults can be attributed to evolving compressive stress field in the Quaternary, where initial NE–SW compression evolved to NW–SE or W–E compression^64–66^.

## Terraces as a monitor of coseismic reverse faulting

The Sendai terraces are not chronologically related to MIS 5e or global sea level changes. Instead, they appear linked to intermittent uplift events associated with the Nagamachi-Rifu fault and associated regional-scale reverse faults. The uplifting of the terraces can be attributed to reverse faulting, indicating that the formation dates of the terrace gravels represent ancient fault activities during the Quaternary. Therefore, the terrace dates provide information about past mega earthquakes associated with these faults. The relative heights of the terraces reflect the amount of vertical displacement caused by the Nagamachi-Rifu fault.

**Figure 6:**
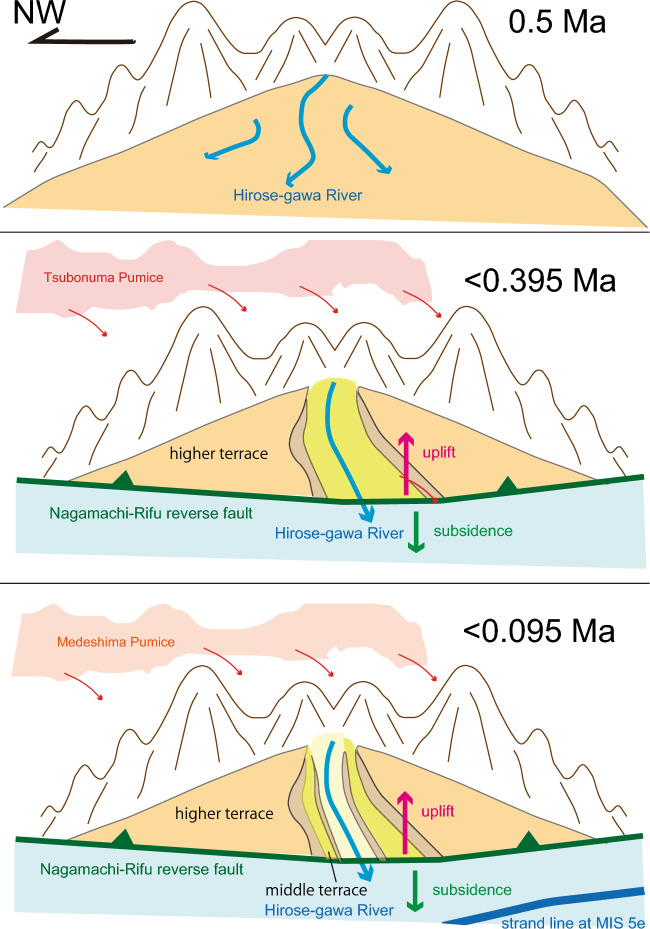
Schematic illustrations of the formative process of the Sendai terraces.

Reverse faulting led to relative subsidence in the footwall, resulting in the deposition of thick fluvial gravels. However, the Ac-Md (and TbP1) pumice fall accumulated on fluvial and hilly areas, as well as the higher and middle terraces of Aobayama and Dainohara (Fig. 6). The strand line of the MIS 5e marine terrace is preserved in the footwall of the Nagamachi-Rifu fault (Fig. 6), indicating that the footwall area experienced subsidence.

The uplift required for terrace formation is associated with reverse faulting that has been active only during the Quaternary. Therefore, the Sendai terraces are restricted to the Quaternary period, and no Pliocene or Miocene terraces have been identified. The trench has been offshore since the middle Cretaceous, but the reason why compression has occurred only within the Quaternary remains unresolved.

**Figure 7:**
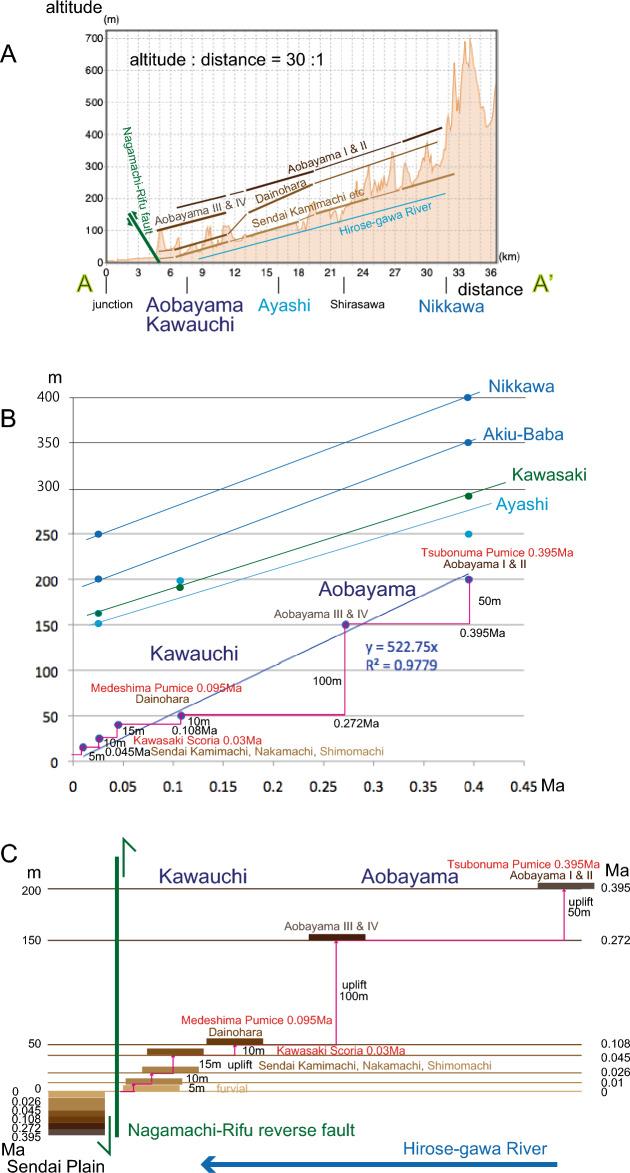
(**A**) Cross-sectional representation. Sendai terraces in relation to the Hirose-gawa River and the Nagamachi-Rifu fault. Injection: the confluence of the Hirose-gawa River and the Natori-gawa River. Section A–A′ indicated on the map, refer to Fig. 3. (**B**) Relationship between Sendai terrace height and age. (**C**) Illustration of Sendai terrace height and age in relation to the Hirose-gawa River and the Nagamachi-Rifu fault.

The estimated emergence times of the terraces are presented in Table 2, and the heights of the terraces (including relative heights) in each representative area are known as shown in map view Fig. 3. Figure 7A shows the relationship between terrace elevations and a trunk stream, which records the Late Quaternary incision of the Hirose-gawa River.

We constructed a diagram depicting the relationship between emergent time and terrace heights (Fig. 7B). The data in the Aobayama-Kawauchi area are complete (Figs. 3, 4E), and the plotted data conform to standard conventions (Fig. 7B). Moving upstream along the Hirose-gawa River and Natori-gawa River from Kawauchi, the altitude increases (Fig. 7A). We plotted the data for the upstream Ayashi, Kawasaki, Akiu-Baba (Fig. 4F), and Nikkawa areas (Fig. 7A). A regression line can be drawn with a gradient of 0.522 mm/year (which represents the tectonic uplift rate calculated by assuming a constant rate and fitting to^67^ with a high coefficient of reliability for the referenced Aobayama-Kawauchi area (Fig. 7A). However, it should be noted that the uplift is not a continuous process but rather occurs intermittently. For example, the hanging wall was suddenly uplifted by 100 m at 0.272 Ma (Fig. 7B; the Nagamachi fault experienced a sudden vertical displacement of 100 m, triggering a mega earthquake). The plots for the upstream areas exhibit similar patterns to the referenced plots (Fig. 7A), and each area documents an abrupt 150 m uplift, likely associated with a mega earthquake event.

Figure 7C represents the geographical expression along the downstream Hirose-gawa River, illustrating that the formation of the Sendai terraces is primarily attributed to activity along the Nagamachi-Rifu fault. The older terraces were uplifted to an altitude of up to 200 m, in contrast to the lowest terrace, which is situated at an altitude of 10 m. Furthermore, the older strata are not stratigraphically covered by the younger strata, which is a typical characteristic of terraces. Conversely, corresponding terrace gravels were rapidly and densely accumulated in a normal order, following the law of superposition in the footwall depression of the Nagamachi-Rifu fault.

## Comments on forecasting earthquakes

A regression line can be drawn with a gradient of ca. 0.5 mm/year to represent the uplifting rate. However, it is important to note that the actual uplifting occurred temporally randomly and intermittently. Since approximately 0.4 Ma, there have been seven major earthquakes, but their occurrence has been sporadic, making it nonsensical to estimate a mean interval. Nevertheless, considering the current subduction zone setting, it is highly likely that the Nagamachi-Rifu fault will become active again, leading to the formation of new terraces and potentially triggering a probable Mw 9 earthquake, similar to the Great East Japan Earthquake of 2011.

## Conclusion

The formation of the Sendai terraces can be attributed to localized and sporadic tectonic uplifts caused by the activity of the Nagamachi-Rifu and related reverse faults. These uplifts have been monitored and observed to identify intermittent fault activities and mega earthquakes. While the mean uplift rate is estimated at ca. 0.5 mm/year, it is not possible to accurately predict the probability of earthquakes or provide precise dates. However, it is important to acknowledge that the potential for future disasters exists.

## Data Availability

The data sets are included in the manuscripts. The data sets used and analyzed during the current study available from the corresponding author on reasonable request.
