# Publisher Correction: The Sendai river terraces monitored the co-seismic mega-thrusting

**DOI:** 10.1038/s41598-023-43275-8

**Published:** 2023-10-03

**Authors:** Soichi Osozawa, Hisatoshi Ito

**Affiliations:** 1 Institute of Geology and Paleontology, Faculty of Science, Tohoku University, Sendai, 980‑8578 Japan; 2 Nuclear Risk Research Center, Central Research Institute of Electric Power Industry, Chiba, 270‑1194 Japan; 3 Present Address: Kawaoso Molecular Bio-Geology Institute, Sendai, 982‑0807 Japan

Correction to: *Scientific Reports* 10.1038/s41598-023-41031-6, published online 28 August 2023

The original version of this Article contained errors in Table 2, where values in the column ‘Age [Ma]^d^’, ‘^206^Pb/^238^U’ for the samples “*TbP1-10”, “TbP1-8”, “TbP1-5”, “TbP1-3”, “TbP1-9”, “TbP1-13”, “TbP1-11(2)”, “TbP1-7” and “TbP1-6(2)”* were omitted. The correct and incorrect columns appear below.

Incorrect:

**Table:** 

Sample name	Age [Ma]^d^
	^206^Pb/^238^U
*TbP1-10*	
*TbP1-8*	
*TbP1-5*	
*TbP1-3*	
*TbP1-9*	
*TbP1-13*	
*TbP1-11(2)*	
*TbP1-7*	
*TbP1-6(2)*	

Correct:

**Table:** 

Sample name	Age [Ma]^d^
	^206^Pb/^238^U
*TbP1-10*	*5.29*
*TbP1-8*	*3.89*
*TbP1-5*	*4.70*
*TbP1-3*	*4.79*
*TbP1-9*	*7.09*
*TbP1-13*	*17.91*
*TbP1-11(2)*	*3.82*
*TbP1-7*	*4.29*
*TbP1-6(2)*	*10.94*

Additionally, Table 3 contained errors, where the columns ‘Column’, ‘Lower tephra’, ‘Age Ma’, ‘Upper tephra’, ‘Age Ma’, ‘Thickness between tepras cm’, ‘Rate mm/year (loam fluvial)’, ‘Loam thickness (maximum) cm’ and ‘Terrace gravel age Ma’ were omitted. The correct and incorrect tables appear below.

Incorrect:

**Table:** 

Terrace alluvium	References	Reference numbers
III Aobayama I and II	Sendai City Board of Education (1990)	^44^
III Aobayama III and IV	Archaeological Research Center on the Campus, Tohoku University (2001)	^45^
II Dainohara	Sendai City Board of Education (2003)	^46^
II Dainohara	Archaeological Research Center on the Campus, Tohoku University (2001)	^45^
II	Hataya (2005)	^47^
Ac-Md	Yagi and Hayata (1989)	^48^
I Sendai Kamimachi	Takeuchi (1986)	^49^
I Sendai Kamimachi	Kosaka et al. (2014)	^50^
I Sendai Kamimachi	Miyagi Prefecture (1997)	^51^
I Sendai Nakamachi	Itagaki et al. (1981)	^24^
I Sendai Shimomachi	Miyagi Prefecture (1997)	^51^
Alluvium	Miyagi Prefecture (1997)	^51^
Alluvium	Kanisawa and Takeuchi (1997)	^43^
Alluvium	Iwanuma City Board of Education (2020)	^52^
Alluvium	Sendai City Board of Education (1989)	^53^
Alluvium	Sendai City Board of Education (2004)	^54^
Alluvium	Awata (2003)	^55^
Alluvium	Awata (2004)	^56^

Correct:

**Table:** 

	Column	Lower tephra	Age Ma	Upper tephra	Age Ma	Thickness between tepras cm	Rate mm/year (***loam*** fluvial)	Loam thickness (maximum) cm	Terrace gravel age Ma	Terrace alluvium	References	Reference number
Aobayama A ruin	Figure 5	On-Pm1	0.095	Za-Kw	0.03	70	***0.010***	300	**0.395**	III Aobayama I and II	Sendai City Board of Education (1990)	^44^
Aobayama E ruin	Figure 5E	Ac-Md	0.095	Za-Kw	0.03	60	***0.009***	160	**0.272**	III Aobayama III and IV	Archaeological Research Center on the Campus, Tohoku University (2001)	^45^
Yamada-Kaminodai ruin	Figure 5F	Ac-Md	0.095	Za-Kw	0.03	50	***0.008***	10	**0.108**	II Dainohara	Sendai City Board of Education (2003)	^46^
Ashinokuchi ruin	Figure 5G	Ac-Md	0.095	Soil	0	100	***0.011***	10	0.105	II Dainohara	Archaeological Research Center on the Campus, Tohoku University (2001)	^45^
Kawasaki	Figure 5C	Aso-4	0.0875	Za-Kw	0.03	60	***0.010***	50	0.133	II	Hataya (2005)	^47^
Adachi		Aso-4	0.0875	Za-Kw	0.03	80	***0.010***	50	0.133	Ac-Md	Yagi & Hayata (1989)	^48^
Sendai Kamimachi		Peat	0.031	Soil	0	180	***0.058***	150	0.056	I Sendai Kamimachi	Takeuchi (1986)	^49^
Sendai Kamimachi	Figure 5H	Za-Kw	0.03	AT	0.0275	30	***0.120***	60	0.350	I Sendai Kamimachi	Kosaka et al. (2014)	^50^
Nagamachi Park No. 1 core	Figure 5I	Peat	0.045		0		0.444		**0.045**	I Sendai Kamimachi	Miyagi Prefecture (1997)	^51^
Sendai Nakamachi		Peat	0.026		0	100	***0.038***	0	**0.026**	I Sendai Nakamachi	Itagaki et al. (1981)	^24^
Nagamachi Park No. 1 core	Figure 5I	Peat	0.009		0	800	0.888		**0.010**	I Sendai Shimomachi	Miyagi Prefecture (1997)	^51^
Nagamachi Park No. 1 core	Figure 5I	Ac-Md	0.095		0	2000	0.210			Alluvium	Miyagi Prefecture (1997)	^51^
Iwanuma Oshiwake core	Figure 5J	Ac-Md	0.095		0	3750	0.394			Alluvium	Kanisawa and Takeuchi (1997)	^43^
Iwanuma Hara ruin		Pottery	0.0014		0	100	0.740			Alluvium	Iwanuma City Board of Education (2020)	^52^
Tomizawa ruin		Peat	0.023		0	700	0.304			Alluvium	Sendai City Board of Education (1989)	^53^
Tomizawa ruin		Peat	0.024		0	400	0.166			Alluvium	Sendai City Board of Education (2004)	^54^
Iwakiri core		Peat	0.0067	Peat	0.0023	1000	2.270			Alluvium	Awata (2003)	^55^
Iwakiri core		Peat	0.008		0	1000	1.250			Alluvium	Awata (2004)	^56^

Furthermore, the caption of Table 3 was incomplete.

“**Table 3.** Estimation of sedimentation rates, tephra ages, and terrace gravel ages (= terrace emergent ages). See references in main text.”

now reads:

“**Table 3.** Estimation of sedimentation rates, tephra ages, and terrace gravel ages (= terrace emergent ages). See references in main text. Significance of bold-italic values: Rate mm/year for loam; significance of underlined values: Rate mm/year for fluvial; significance of bold values: Adopted terrace gravel age Ma.”

The original Article has been corrected.

